# Exploring the benefits and challenges of Internet of Things (IoT) during Covid-19: a case study of Bangladesh

**DOI:** 10.1007/s43926-021-00020-9

**Published:** 2021-11-18

**Authors:** Nahida Sultana, Marzia Tamanna

**Affiliations:** 1 Department of MIS, Bangladesh University, Dhaka, 1207 Bangladesh; 2 Department of MIS, East West University, Dhaka, 1212 Bangladesh

**Keywords:** IoT, Bangladesh, COVID-19, Internet of Things

## Abstract

The Internet of Things (IoT) is expected to have a huge impact, especially during the pandemic period. The study reveals that people are using the IoT mostly for education purposes (as students and educators), office work, banks and medical purposes during the pandemic. The topmost benefit of using IoT services experienced by people during pandemic situations is that it helps to strictly maintain physical distance. However, the greatest challenge faced by people is that the use of the IoT increases social distancing and reduces personal communication. Data were collected through a questionnaire distributed online and using a convenient random sampling method. A total of 260 participants’ properly completed responses were analyzed after conducting Three-fold validation. Research method was quantitative and empirical. Although, some studies have been found about IoT prospects in Bangladesh, no study has specifically explored the benefits and challenges of IoT services in diverse fields of Bangladesh during this new normal COVID-19 situation. The results can be beneficial to academic scholars, business professionals and organizations in different sectors and any other parties interested in determining the impact of IoT services on pandemic.

## Introduction

The Internet of Things (IoT) is a new paradigm move in the IT (Information Technology -usage of computer networks for communicating information) arena. The term “IoT”, which is elaborately known as “internet of things”, has been coined from two words—‘internet’ and ‘things’. The internet is a universal system of interrelated networks of computers that use Internet Protocols (IPs) and Transmission Control Protocols (TCPs) to serve billions of users around the world [24]. In the IT world, the internet of things is one of the hyped concepts around the world. The term ‘Internet of Things’ has appealed attraction through the projection of worldwide infrastructure of connected physical objects, facilitating anytime, anyplace networking for anything [22]. This can also be considered a universal network that allows communication between humans to humans, things to things and humans to things through a unique identity to each object [1]. Ghasempour [11] stated that Internet of Things (IoT) is the connection between people and things in any location, with anyone and anything using any network or service.

This term is sixteen years old, but the idea of a connected device is longer than this. Earlier, this idea was termed “pervasive computing”. Kevin Ashton pioneered the actual term “Internet of Things” in 1999 at Proctor & Gamble at the time of his work. Working in supply chain management, Kevin desired attracting senior management to one new exhilarating technology named RFID. He termed his presentation the “Internet of Things” as in 1999, the Internet was the newest trend [9].

**Fig. 1:**
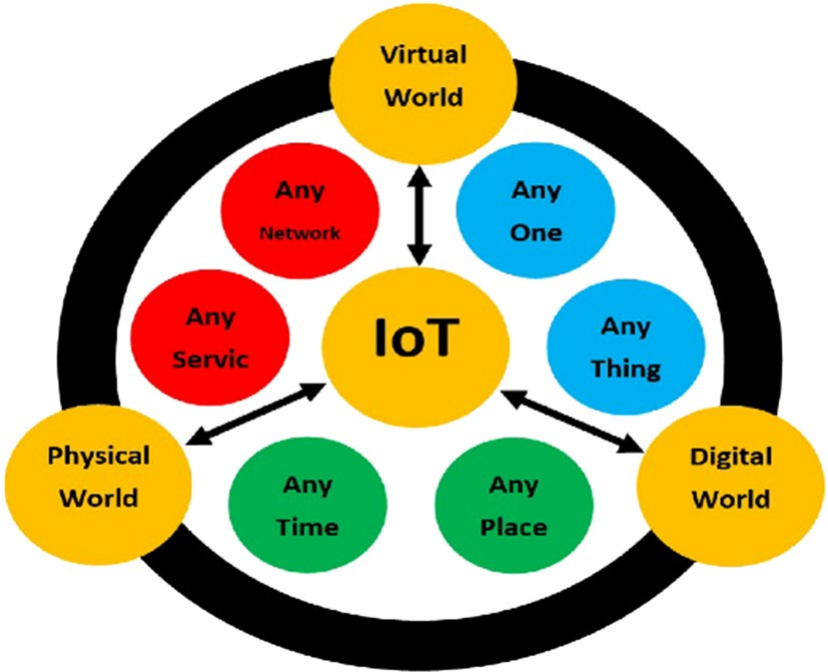
IoT Concept Ghasempour [11]

**Fig. 2:**
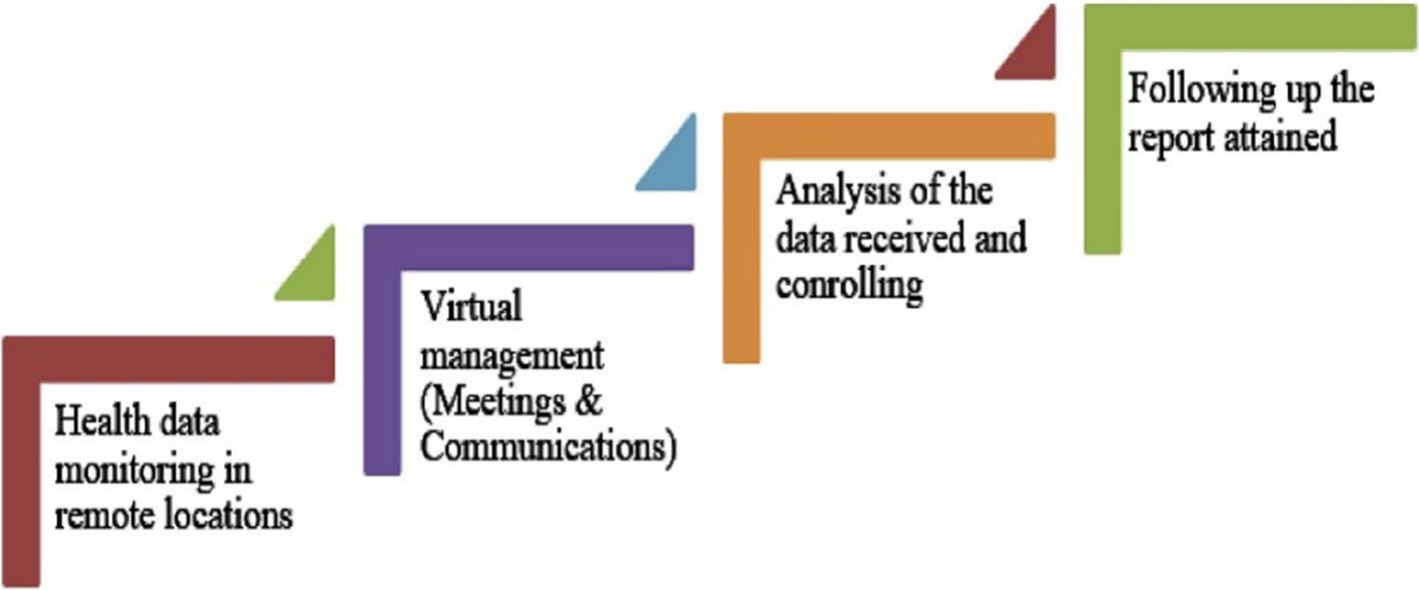
Processes involved in IoT for fighting Covid-19 in hospital management [38]

The application of the Internet of Things is in many areas. The application begins with the automation of home to wearable objects. Ghazaleh and Zabadi [12] provided significant insight on the new frontier of Customer Relationship Management (CRM) focusing on the use of IoT in Bangladesh. Moreover, they contributed to provide effective process of implementing IoT in connecting business and customer. Singh et al. [38] focused on automated and transparent treatment process to tackle challenges during Covid-19. A process (Fig. 1) has been revealed that shows step-up process for executing IoT in healthcare system. Couple of advantages of using IoT (Fig. 2) have been identified in medical system.

Bakla [2] made distinction between the use of IoT at the management and instructional levels in the educational contexts and revealed a great challenge of using IoT in education is digital divide and benefits include using learner analytics. In this situation of COVID-19, applications supported by IoT tools are used to minimize the spread of coronavirus by observing the patients, performing primary analysis and following the specified protocols after the recovery of patients [28]. In Bangladesh, it is also practicing everywhere during COVID-19. The revolution of the IoT is reshaping the modern healthcare system with economic, technological and social incorporation. Through connection with the internet of things, the healthcare system is evolving from convention to a more customized healthcare system enabling easy diagnosis, treatment and monitoring of patients [28]. Apart from direct application in healthcare, it is also being used as wearable technologies, remote working, drones, robots, retail operations, data storage and security devices, collaboration tools, distant banking, e-commerce etc. As a developing country, Bangladesh is facing both opportunities and challenges in using Internet of Things services during the pandemic situation. In COVID-19, the uses of Internet of Things in different sectors in Bangladesh are known. Then, the benefits and limitations experienced in using the services by the IoT are to be examined, which enable us to know about the opportunities and challenges of IoT devices in Bangladesh. After that, attitudes of people toward using IoT services can be found. Particularly, the following research questions have been addressed:

RQ1: What are the uses of IoT services in different sectors and in Bangladesh during COVID-19? What are the kinds of usage of IoT services? Overall, what are the users’ perspective?

RQ2: What are the benefits experienced in using the IoT during COVID-19?

RQ3: What are the challenges experienced in using the IoT during COVID-19?

### Research objectives

This research aims to understand the opportunities and challenges of the Internet of Things in Bangladesh during COVID-19. Specific objectives include:

- To examine the uses of IoT services in different sectors in Bangladesh during COVID-19.
- To discover the benefits experienced in using IoT during COVID-19
- To identify the challenges experienced in practicing IoT during COVID-19.

## Literature review

The term “Internet of Things” (IoT) indicates an advanced technology that links all smart objects without any human interactions in a network. In a wide variety of industrial and academic disciplines, it is a new research topic that has received substantial convincing research ground in recent years [26]. Walcott [41] stated that by many governments, modern digitalized defense strategies have been implemented in the war against COVID-19. Digital technologies and innovations have gradually become the humanity’s strongholds during this time. At a certain pace and scale, the deployment and creation of emerging digital technology are strongly motivated by the economic needs posed by COVID-19. Digital tools are being used to promote the public health response to COVID-19 internationally including touch tracking, population monitoring, action assessment and event recognition focused on engagement with the public and mobility of data.

According to Islam [19] the related advance technology and integrated position of the IoT has the potential to be a significant advancement in attempts to control the emerging pandemic. To achieve SDG’s (sustainable and digitalization goals), the potential of the IoT would have a major impact in the developed world. Practice of stated protocols, surveillance of patients and primary detection process are necessary to minimize the possible spread of the coronavirus, where IoT enabled applications and devices are used.

Javaid [20] stated that information and physical objects with the internet can be received and sent through the internet of things (IoT). Wireless and fixed internet controlled the smart hospital devices and concepts. In the medical field, various medical devices, diagnostics, sensors, advanced imaging devices and artificial intelligence are central to implementing the IoT. To accomplish the required task smart devices can share data and capture it in daily life. This application is reaching homes, smart cities, entertainment systems and cars and connected healthcare. In both old and new societies and industries these advances devices improved the quality and productivity of life. During the COVID-19 pandemic, this technology is booming in monitoring healthcare.

Nasajpour et al. [28] added that to provide proper attention, this technology stored all COVID-19 patient-related information in the cloud, which can further help. Without any human interaction, the IoT interrelates all mechanical, digital and computing technologies to transfer the data through the internet. In this critical scenario, due to untimely and improper information about health, many people die. The IoT captures the daily activity of a person and alters the health problem, it uses sensors to quickly notify the system about health-related issues [8].

To ensure successful operation in the medical field there is an essential requirement for proper equipment. During the COVID-19 pandemic, the application of IoT gives better care to the patient. Via smart connecting devices, smart medical devices are linked to transfer essential health facts to physicians. These devices, with the help of the IoT, monitor real-time data successfully to save lives from different health problems. The IoT has a high capability to analyze and operate successful operations and after services [20].

According to Müller [27], the IoT is a cyber-physical system without any human intervention in which digital and mechanical machines exchange data in a network. In consumer applications and corporations, such systems use real-time analytics, robotics, cloud computing, sensors and machine learning to fully automate the processes. In this current crisis, many companies have the chance to rethink their business process and press the reset button. Smart manufacturing automated and connected assembly lines can exchange data, allow for shorter cycle times, decreased order times, lower inventory levels, higher production flexibility, predict events and autonomously decide how to deal with problems. In addition to large caps in the long run, it also boosts productivity growth [8].

In the trending industry smart homes are another trending one. To improve traffic and waste management, the IoT has long experienced networks and flood and fire sensors for making houses safer. The COVID-19 situation speeds up the deployment of smart devices. To detect fevers, infrared cameras were used in public spaces such as train stations and airports. To examine whether large offices abide by social-distancing measures, software can be used to analyze video feeds [31].

For the adoption of this interconnected technology, those who provide the services and the infrastructure are mostly contact-free and from this economy are likely to benefit. To oversee and coordinate the factory processes, the IoT includes data storage and data security as well as cloud systems. The hardware ranges from assistive industrial robots and 3D printing for additive manufacturing to sensors and semiconductors [29].

Israel et al. [16] indicated that in 2020, we have seen the widespread use of cloud computing and collaboration platforms, as many of us have taken remote work. In 2021, remote monitoring systems will become more commonplace where physical presence is necessary. When an issue requires human interaction in industrial operations, staff are able to do so remotely and can monitor the automated manufacturing processes either in-person or remotely.

In this COVID-19 pandemic, the IoT even advanced the online retail industry. Evangelos et al. [7] stated that giant merchants and smaller merchants keep their doors open virtually. To minimize human contracts in automated supermarkets and distribution centers, large companies such as Walmart and Amazon engage in all kinds of automation, including robotics. Some already used technologies such as radio frequency identification (RFID) help to continue contact-free payment. It is a common way to track the inventory in the retail industry to improve shoppers’ in-store experience, and it is used to collect data on customers’ purchases. Some apps from Samsung, Google and Apple help to grow cashless and contact-free payment options [41].

According to UNDP [39], to measure the contribution added of emerging technology to the pandemic response, COVID-19 is underway. The IoT has some response support tools for this pandemic. Culture, climate and the economy are the key factors that are shaped by digitalization and sustainability. Both the growth smart of new digital technology and the emerging COVID-19 pandemic have effectively systemized the equation. Few scholars have considered achieving SDGs in emerging regions in this pandemic to be the ability of the IoT.

Modern healthcare systems, economics, incorporating technological and social prospects. Have reshaped the revolution of the IoT. IoT devices can be connected to the transferring data and to the internet for further monitoring. In this COVID-19 situation, due to advanced user experiences, a better quality of services and lower expenses, the IoT has increasingly become a vital technology for the healthcare systems. More technologically advanced healthcare systems provide more personalized systems that help treat patients remotely, diagnosed and monitored easily. As of the last report on 29th March 2021, WHO showed globally 126,359,540 confirmed cases of COVID-19 included 2,769,473 deaths [42]. In Bangladesh, according to the DGHS press release, there were 595,714 confirmed cases of COVID-19 and 8,904 deaths confirmed by RT-PCR [6].

Radanliev and De Roure [32, 33] focused on the IoT ethical design and IoT design update but didn’t talk over the effects of coupled and multifaceted risks from IoT system. Prior to combination of new IoT technologies, they find out that supply chains must be attached with ethical consideration of the cyber risks. On the security of vaccine supply chain’s introduction of IoT brings greater risk and in this paper, they help the medical organizations, governments and healthcare practitioners on developing a framework to provide guidance [34].

Based on the digital humanities tools this paper offered an abstract epistemological framework during the Covid-19 Period. This epistemological framework will help the developing countries connecting the value of new technology solutions. From the Covid-19 research on qualitative data samples, studying narratives, case study and grounded theory this epistemological framework is designed [32, 33]. In the past year and future, the role of the IoT in the COVID-19 pandemic has been vital. To help fight the disease, IoT applications such as health monitoring wearables, contact tracing devices, temperature sensors, thermal cameras and parcel tracking provide the critical data needed to safely distribute the COVID-19 vaccines according to the [10]. To provide safe remote access to industrial machines and to promote social distancing, the IoT has helped make automated activities in warehouses and more resilient supply chains in this COVID situation.

Parvez et al. [30] represented a conceptual framework model through exploring the prospects of Internet of Things of Bangladesh socio-economic development sectors and the model showed that Bangladesh needs to develop a set of guidelines for the adoption of IoT to implement national IoT strategy. Miazi et al. [25] presented IoT in developing countries like Bangladesh, and revealed the opportunities such as workplace safety, road safety, mHealth, environment monitoring and utilities management and challenges include technical challenge, device reliability, financial challenge, and security and privacy issues. Sarker et al. [37] presented some best potential applications, challenges, and future opportunities in the area of internet of things.

Based on the literature review found regarding IoT applications in Bangladesh, no such a complete work has been found regarding the present scenario of using IoT in different sectors during Covid-19. Hence, to portray the impact of Internet of Things applications in different sectors during the pandemic, a conceptual model was created. This study examines the benefits and challenges experienced with the adoption of IoT services in different sectors during the pandemic, and the results would help organizations respond and adjust quicker growth to IoT services and achieve greater competitive advantage.

## Research methodology

This Section aims at discussing how questionnaire was developed for collecting data, how many sections are there, validation of questionnaire data accuracy, methods of sample collection, parameter, analytical method of collected data, demographic profile of respondents and reliability and validity testing of research instruments.

### Instrument development

Primary data for this study were collected using a structured questionnaire. The survey consisted of three major parts in addition to demographic information. The demographic information section consists of four main questions: participants’ age, level of education, gender and occupation. Then, the next section, part 1 includes questions about the current field participants working and kinds of IoT service experiences by the participants. The participants were asked to share their perception by rating their responses on a five-point level of agreement Likert scale. Part 2 of the questionnaire consists of 12 benefits experienced while using IoT services during COVID-19. Twelve related statements were used in the survey, as shown in Table 3. Finally, Part 3 of the questionnaire consists of 12 challenges experienced while using IoT services during COVID-19. Twelve related statements were used in the survey, as shown in Table 4.

### Sample and data collection

The data for this study were collected during May and June 2021 through a structured questionnaire distributed online. The sampling method was convenient sampling, where IoT users in different fields were identified in a convenient approach. Threefold validation was conducted for checking questionnaire data accuracy. The results were 0.8012, 0.7024, 0.7236. The mean of accuracies of threefold is 0.7424, which means 74% of the overall accuracy.

Approximately 500 questionnaires were distributed online to the respondents which included 70% male and 30% female; as males are more active in different sectors in the country. A total of 310 questionnaires were returned, and among them, 260 fully completed surveys were received, resulting in a response rate of approximately 53%. Among respondents, there are 65% male and 35% female. In the age parameter, most of respondents (53%) belong to the 26–35 age range.

### Analytical method

To analyze the collected data in this research, IBM Statistical Package for Social Science (SPSS) version 28 was used. First, the reliability and validity of the data constructs were tested. To examine the consistency and reliability of each construct, Cronbach alpha values and corrected item-total correlation were used. To inspect the construct validity, an exploratory factor analysis was also used for each of the items within the constructs. Second, descriptive analyses were conducted to compute the frequency means for each item within constructs where a five-point Likert scale of agreement was used. Some graphical presentations with percentage analysis have also been performed.

### Respondents’ demographic profile

The demographic profile of the respondents is given in Fig. 4 and Table 1.

**Table 1:** Profile of the participants

Question	Frequency (n = 260)	Percentage
Gender Male Female	170 90	65 35
Education Bachelors Masters PhD	118 127 15	45 49 6
Field and area of use Medical Wearable devices Workplaces Education Merchandise Bank Smart Home	39 32 95 167 24 74 41	15 12 36 64 9 28 15

#### Age

From Fig. 2, we can interpret that the majority of the respondents were below the age of 35. Twenty-one percent of male people were below 25, 31% were below 35 and only 5%, 3% and 2% of male participants were below 45, below 55 and 55 or above the age category respectively. However, 12% of female respondents were below 25 years old, 19% were below 35 years old and only 4%, 2% and 1% of female participants were below 45, below 55 and 55 or above 55 years old, respectively.

#### Gender

Table 1 indicates 65% male participants and 35% female participants.

#### Education

In Table 1, among the 260 participants, 45% belonged to a bachelor’s degree, 49% belonged to masters and 6% belonged to a PhD.

#### Field of expertise

In terms of field expertise, the participants were asked to select fields of area (more than one field) they were using IoT services. Table 1 shows that the majority of the participants (64%) belonged to the education field, 36% used IoT services in workplaces, 28% in Bank, and 15% were in the medical field.

### Reliability and validity of research instrument

Reliability denotes an indicator of evaluating the consistency of the scale items within a construct [5, 18]. Reliability testing ensures the degree to which the research instruments are error-free. Cronbach’s alpha (α) is a common measure of scale reliability and internal consistency of the items [40]. Cronbach alpha can be computed through correlating score of each scale with total score of each observation and then comparing that to the variance of individual item scores [14].

To measure the consistency and reliability of the two constructs of this study, Cronbach’s alpha value, Cronbach's alpha if item deleted and corrected item-total correlation were used. As shown in Table 2, Cronbach’s alpha coefficient value for the benefits of using IoT services was 0.832, and for the challenges of using IoT services, it was 0.808. Cronbach’s values exceeding the alpha coefficient of 0.7 deliver reliability evidence for the internal homogeneity of measurement scales [35].

**Table 2:** Summary of the findings of reliability test

Construct	No. of items	Mean	Cronbach Alpha	Range of Cronbach's Alpha if Item deleted	Range of corrected item-total correlation
Benefits in using IoT services	12	3.896	0.832	0.810–0.840	0.407–0.617
Challenges in IoT services	10	3.595	0.808	0.780–0.813	0.427–0.586

Kemp [21] suggested that the closer to 1 the value is, the more reliable the construct is. As the Cronbach’s alpha coefficient values for the two constructs in this study are well above 0.8, it can be concluded that the survey instrument is an extremely reliable research tool. Cronbach’s alpha if one item from the constructs was deleted was also calculated, and the ranges were 0.810–0.840 and 0.780–0.813, respectively, which were above the standard value of 0.70. The corrected item-total correlation values were also computed for all items in the two constructs. In Table 3, except for the 1 item (corrected item-total correlation = 0.246) in the first construct used, 11 items’ item-total correlation values ranged from 0.407 to 0.617. In Table 4, in the second used construct, 9 items’ item-total correlation values ranged from 0.427 to 0.586, excepting for only 1 item (corrected item-total correlation = 0.284). Gliem and Gliem [13] suggested that item total correlated values should be at least 0.40. As the corrected item total correlations of the maximum items (20 items) from 22 items in two constructs are above 0.40, it indicates good internal consistency among most of the used scale items.

**Table 3:** Participants’ perceptions about the benefits experienced while using IoT

Items	Means	Factor loading	Item-total correlation	Rank
It helps to strictly maintain physical distance	4.20	0.652	0.547	1
It saves time	4.18	0.626	0.535	2
Easy to use	3.98	0.625	0.521	3
Easy to access information	3.97	0.706	0.588	4
Facilitates the communication	3.93	0.634	0.513	5
It allows contactless work	3.90	0.653	0.246	6
Reduces manual job	3.90	0.506	0.407	7
Facilitates location-based services	3.80	0.584	0.481	8
It saves cost	3.80	0.575	0.482	9
Services are available at any time	3.75	0.634	0.536	10
Services are convenient	3.74	0.720	0.617	11
Ensures security in transaction/data processing	3.59	0.559	0.457	12

**Table 4:** Participants’ perceptions about the challenges faced while using IoT services

Items	Means	Factor loading	Item-total correlation	Rank
It increases social distancing	3.98	0.753	0.284	1
It reduces personal interaction	3.85	0.576	0.503	2
Mounting number of frauds	3.78	0.583	0.462	3
Technological complications	3.66	0.696	0.578	4
Lesser jobs/loss of jobs	3.58	0.539	0.427	5
Unauthorized access to data	3.54	0.688	0.556	6
Mobility challenges	3.53	0.712	0.586	7
It compromises data privacy	3.50	0.577	0.448	8
Lack of flexibility	3.32	0.715	0.581	9
It seems complex	3.21	0.576	0.453	10

To examine the validity of the constructs, exploratory factor analysis (principal component) was used. According to Hair et al. [17], values of factor analysis higher than 0.30 are considered significant, values higher than 0.40 should be considered more significant and values greater than 0.50 or above should be taken very significant. As shown in Table 3, the benefits experienced while using IoT services construct-generated factor loading values ranged from 0.506 to 0.720. As shown in Table 4, the challenges faced while using IoT services construct-generated factor loading values ranged from 0.539 to 0.753. Thus, the construct validity of the instruments used in the study is very significant.

## Result analysis

### Usage of Internet of Things (IoT) in different sectors

**Fig. 3:**
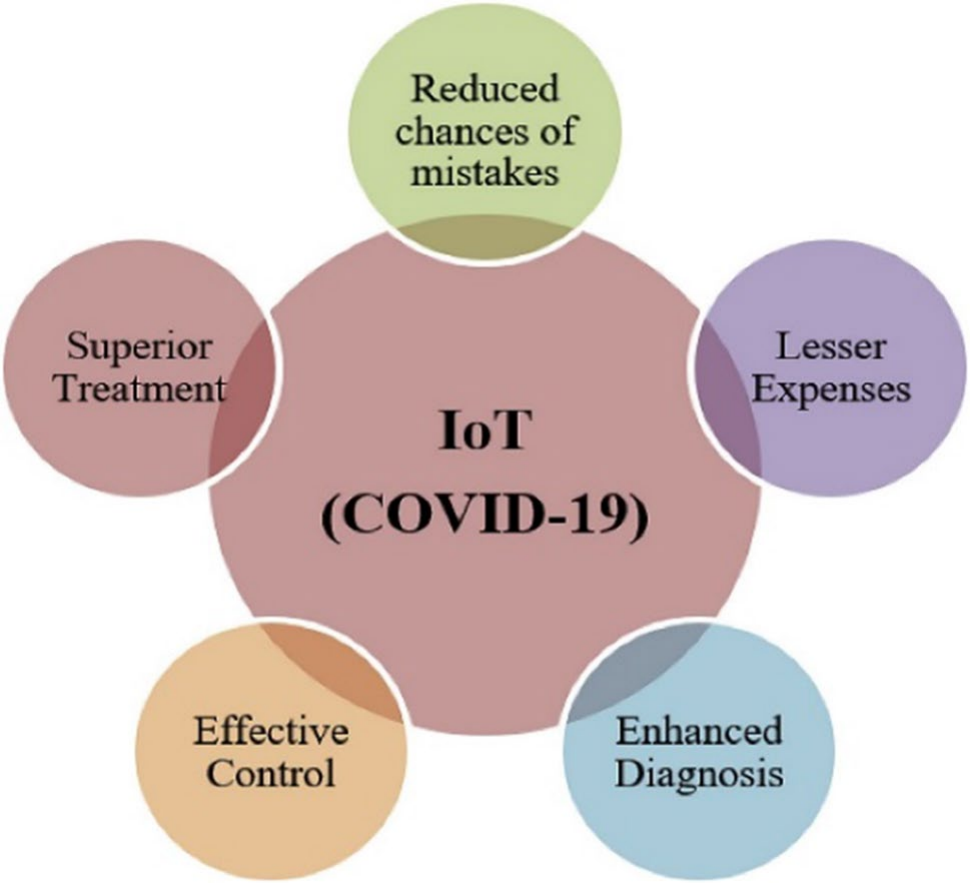
Major merits of using IoT [38]

Participants in the study were asked to select the uses of Internet of Things (IoT) services in diverse sectors. Figure 3 shows that the largest population (64%) uses IoT services during COVID-19 in the education sector. Approximately 36% users use IoT in the offices. A notable number of users are in the banking sector (28%) and medical sector (15%).

### Kind of users

**Fig. 4:**
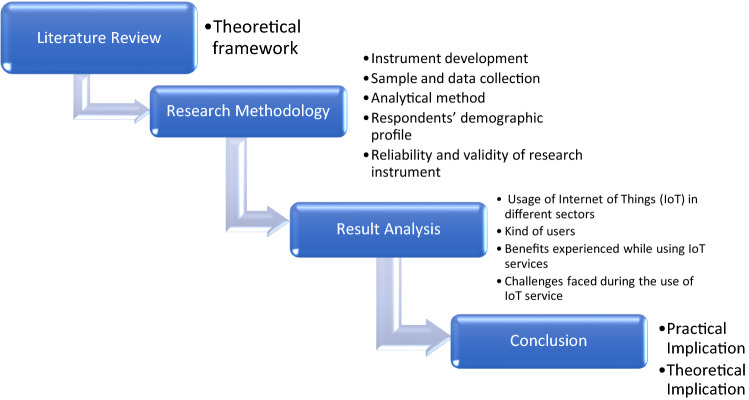
Organization of the study

**Fig. 5:**
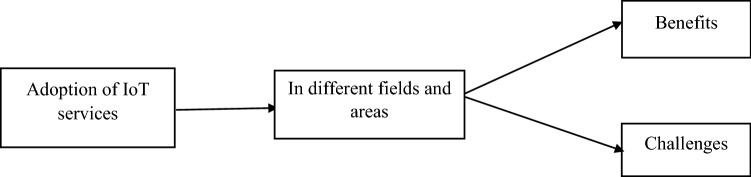
Research Framework

**Fig. 6:**
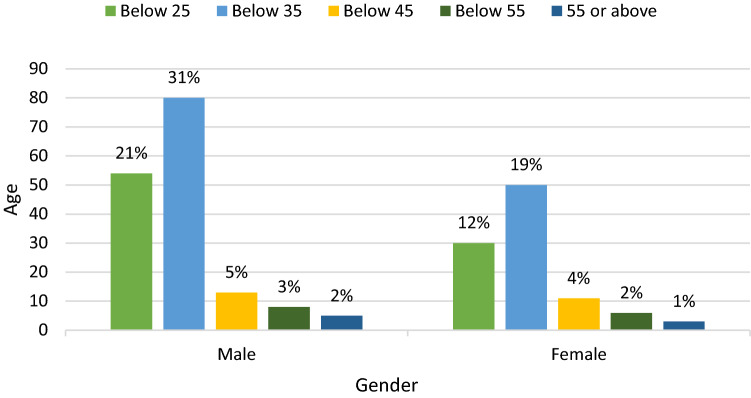
Participants’ age

**Fig. 7:**
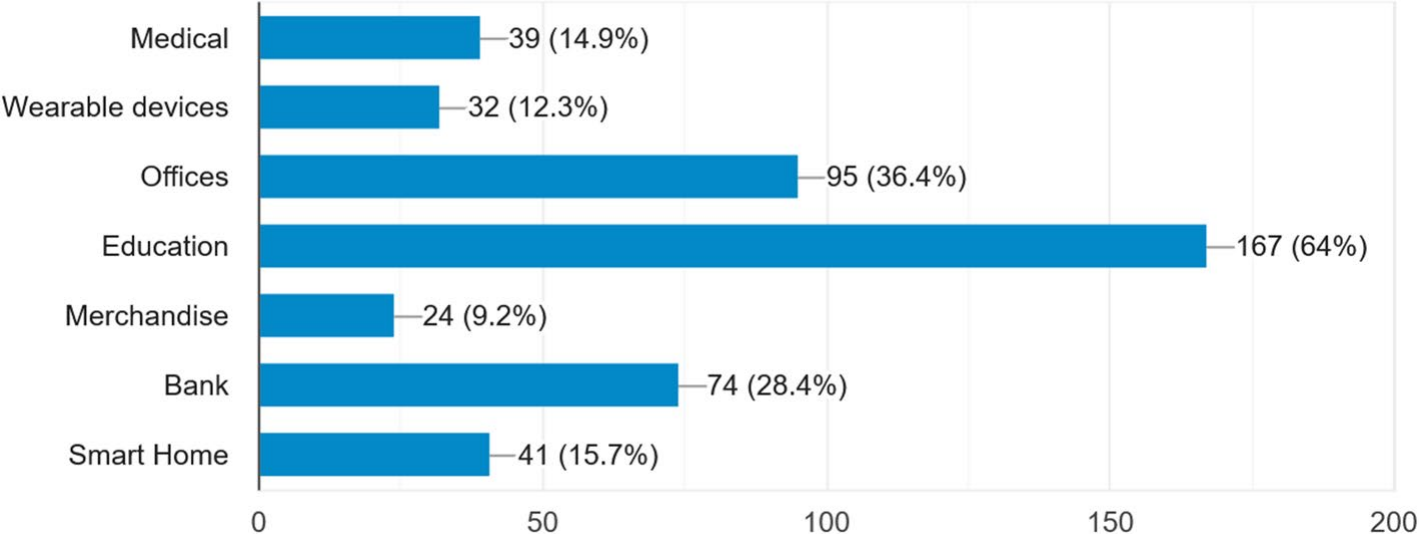
Users of the Internet of Things (IoT) in COVID-19

**Fig. 8:**
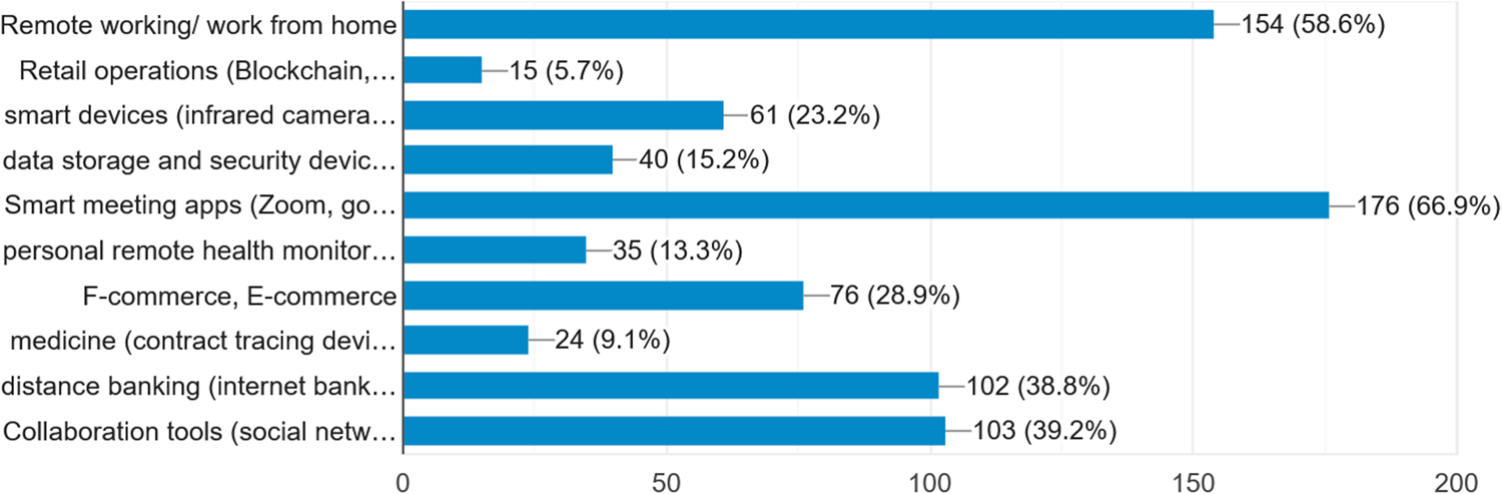
Kind of IoT service users

Participants of the study were asked to say about the kind of IoT services they are using in the stated fields. Figure 4 demonstrates that the majority of the population (67%) uses smart meeting apps. A remarkable number of people (59%) do remote working/work from home. Another significant portion of users are in distance banking (39%) and collaboration tool use (39%). During the pandemic, a new kind of user emerges in F-commerce and E-commerce contributing appx. 29% of the whole. However, a very small number of people are involved in retail operations (6%) (Figs. 5, 6, 7, 8).

### Benefits experienced while using IoT services

The constructs used to study the benefits experienced while using IoT services includes 12 items (Table 3). The respondents were asked to rate these 12 items using a five-point Likert scale ranging from “1 = Strongly Disagree” to “5 = Strongly Agree”.

The results, as shown in Table 3, indicate that the topmost benefit experienced in using IoT services is “*It helps to strictly maintain physical distance”* (Mean = 4.20). This result supports some theoretical benefits indicated in several studies regarding the importance of using the IoT in maintaining physical distance during COVID-19 [8, 27, 29, 31]. The second top benefit experienced in using IoT services is *“It saves time”* (Mean = 4.18). This finding supports the theoretical and empirical benefits pointed out in several studies regarding saving time in terms of shorter cycles, decreased order times and real-time analytics [20, 27]. The next benefits in IoT are in third and fourth ranks “*Easy to use* (Mean = 3.98)” *and “Easy to access information* (Mean = 3.97)”, respectively. These findings support the findings of several studies related to health diagnosis and data access [27, 28]. The fifth benefit experienced was “*Facilitates the communication*” (Mean = 3.93).

Conversely, the least five benefits experienced in IoT services during the pandemic include *facilitates location-based services* (Mean = 3.80), *saving costs* (Mean = 3.80), *services being available at any time* (Mean = 3.75), *services being convenient* (Mean = 3.74) and *ensuring security in transaction/data processing* (Mean = 3.59). The lowest rated IoT benefit is “*Ensures security in transaction/data processing* (Mean = 3.59). Very few studies have been found regarding data security. During the pandemic, fraudulence and violation of data privacy are on rise. Therefore, more attention is required to ensure the security of data processing.

Factor loading is used to validate research construct as discussed in 3.5. Item-total represents correlation between each item and total scale score. For exploratory study, 0.20 is acceptable for item-total correlation [3]. In this study, values of items-total correlation are well above 0.20, which denotes good correlation between each item and total score.

### Challenges faced during the use of IoT service

The construct used to examine the challenges experienced while using IoT services consists of 10 items (Table 4). The results, as shown in Table 4, reveal that the highest ranked challenge faced while using IoT services during COVID-19 is “*increasing social distancing”* (Mean = 3.98). Most of the people agreed that the use of IoT is increasing social distancing. This top challenge supports the findings regarding IoT use prone to social distancing [10, 31]. Second, “*reducing personal interaction”* (Mean = 3.85) has been seen as another key challenge in using IoT service use in pandemics. Many people said that the use of IoT has been decreasing personal connections with others which is one of the major challenges to them.

Conversely, the two least ranked challenges in using IoT services during the pandemic include “*Lack of flexibility*” (Mean = 3.32) and “*It seems complex”* (Mean = 3.21). As people are becoming more adapted to this new normal situation of COVID-19, so are technologically adaptable. Although, hundreds of studies have been published covering the technological and technical challenges to IoT services [4, 15, 23, 36], these findings clearly oppose this. People are facing fewer challenges in using IoT in terms of flexibility and complexity.

Moreover, Factor loading is used to validate research construct as discussed in 3.5. Values of items-total correlation are above 0.20 as well, which denotes good correlation between each item and total score.

## Conclusions

In the context of the current new normal situation, activities without human interaction are increasingly driven by Internet of Things (IoT) services. The study reveals the benefits and challenges experienced by people in diverse sectors. The results show that younger people (below 35 years of age) are more likely to use Internet of Things services in different sectors. During the pandemic, people are using the IoT mostly for education purposes (as students and educators), office work, banks and medical purposes. The topmost benefit of using IoT services, experienced by people during pandemic situations is that it helps to strictly maintain physical distance. However, benefit regarding security is the least rated benefit experienced by people. The greatest challenge faced by people is that the use of the IoT increases social distancing and reduces personal communication. However, people now face less challenge in using IoT services in terms of inflexibility and complexity. The findings of our study have both theoretical and practical implications. Further research can be conducted by extending the variables considered here and investigating peoples’ intention towards using IoT.

### Theoretical implication

This study provides a foundation for future researchers to study the users’ experiences in using IoT tools in different fields during COVID-19. There are very few studies found regarding IoT service experiences in Bangladesh during pandemic crisis. This study will assist in finding the benefits and challenges faced by IoT users in diverse sectors during the pandemic. Further research can also be possible by increasing the sample size including the rural population, which might reflect the entire scenario of customers’ experiences regarding IoT services in this pandemic time. Furthermore, more variables can be considered in future research.

### Practical implication

Our study helps the organizations adjust quickly and respond in a timely manner to the growth of IoT services and it also helps to examine the challenges and benefits of adopting the IoT in different sectors. To emerging countries such as Bangladesh the findings act as a realistic roadmap for applying IoT technologies. Instead of reducing work opportunities, the use of the IoT creates simpler work practices and more jobs. In the field of hospitals, financial organizations, education, utilities and others areas, the IoT has enabled improvements to significant facilities which gives a modern scheme to technology advancement. The results may help the other sectors of Bangladesh learn lessons from the experience of the case institutions. The use and impact of the IoT in different sectors during the pandemic will benefit further application and will increase the usage of technology.

## Data Availability

The datasets generated before the analyses or the datasets used during analyses have been attached to “the related file option” in the name “IoT data”.

## References

[1] Aggarwal R, Das ML. RFID security in the context of" internet of things". In: Proceedings of the First International Conference on Security of Internet of Things. 2012. p. 51–6.

[2] Bakla A (2019). A critical overview of Internet of Things in education. Mehmet Akif Ersoy Üniversitesi Eğitim Fakültesi Dergisi.

[3] Cristobal E, Flavian C, Guinaliu M (2007). Perceived e-service quality (PeSQ): Measurement validation and effects on consumer satisfaction and web site loyalty. Manag Serv Qual.

[4] Da Xu L, He W, Li S (2014). Internet of things in industries: A survey. IEEE Trans Industr Inf.

[5] DeVellis RF, Thorpe CT (2021). Scale development: Theory and applications.

[6] DGHS. Coronavirus (Covid-19). DGHS: https://dghs.gov.bd/index.php/en/component/content/article?id=5393

[7] Evangelos AK, Nikolaos DT, Anthony CB (2011). Integrating RFIDs and smart objects into a unified internet of things architecture. Adv Int Things.

[8] Fahrni S, Jansen C, John M, Kasah T, Körber B, Mohr N (2020). Coronavirus: Industrial IoT in challenging times.

[9] Forbes. Internet of Things. http://www.forbes.com/sites/jacobmorgan/2014/05/13/simple-explanation-internet-things-that-anyone-can-understand/#22629edf6828. 2014. Accessed 31 May 2021.

[10] Forum WE. State of the Connected World. Retrieved from 2020 Edition: http://www3.weforum.org/docs/WEF_The_State_of_the_Connected_World_2020.pdf. 2020.

[11] Ghasempour A (2019). Internet of things in smart grid: Architecture, applications, services, key technologies, and challenges. Inventions.

[12] Ghazaleh MA, Zabadi AM (2020). Promoting a revamped CRM through Internet of Things and Big Data: an AHP-based evaluation. Int J Org Anal..

[13] Gliem J. Gliem R. Calculating, Interpreting, and Reporting Cronbach’s Alpha Reliability Coefficient for Likert-Type Scales. In 2003 Midwest Research to Practice Conference in Adult, Continuing and Community Education. Columbus, OH. 2003.

[14] Goforth C. Using and Interpreting Cronbach’s Alpha. University of Virginia Library. https://data.library.virginia.edu/using-and-interpreting-cronbachs-alpha/. 2015.

[15] Haddud A, DeSouza A, Khare A, Lee H (2017). Examining potential benefits and challenges associated with the Internet of Things integration in supply chains. J Manuf Technol Manag.

[16] Haim Israel LK-T. Covid-19 Investment Implications Series:The World After Covid Primer. Thematic Investing, 1–16. 2020. https://www.bofaml.com/content/dam/boamlimages/documents/articles/ID20_0467/the_world_after_covid.pdf

[17] Hair JF, Black WC, Babin BJ, Anderson RE, Tatham R (2006). Multivariate data analysis.

[18] Hinkin TR (1995). A review of scale development practices in the study of organizations. J Manag.

[19] Islam A, Anum K, Dwidienawati D, Wahab S, Abdul Latiff A (2020). Building a post COVID-19 configuration between Internet of Things (IoT) and sustainable development goals (SDGs) for developing countries. J Arts Soc Sci.

[20] Javaid M, Khan IH (2021). Internet of Things (IoT) enabled healthcare helps to take the challenges of COVID-19 Pandemic. J Oral Biol Craniofac Res.

[21] Kemp F. Applied multiple regression/correlation analysis for the behavioral sciences. 2003.

[22] Kosmatos EA, Tselikas ND, Boucouvalas AC (2011). Integrating RFIDs and smart Objects into a unified in-ternet of things architecture. Adv Int Things: Sci Res.

[23] Lee I, Lee K (2015). The Internet of Things (IoT): Applications, investments, and challenges for enterprises. Bus Horiz.

[24] Madakam S, Lake V, Lake V, Lake V (2015). Internet of Things (IoT): A literature review. J Computer Commun.

[25] Miazi NS, Erasmus Z, Razzaque A, Zennaro M, Bagula A (2016). Enabling the internet of things in developing countries: Opportunities and challenges. 2016 5th International Conference on Informatics, Electronics and Vision (ICIEV).

[26] Mohammed T, Jean-Yves C, Peter B, Christophe R (2020). Petrogenesis of the post-collisional Bled M’Dena volcanic ring complex in Reguibat Rise (western Eglab shield, Algeria). J Afr Earth Sci.

[27] Müller J. COVID-19 AND THE INTERNET OF THINGS. 2020. DWS: https://www.dws.com/insights/cio-view/macro/internet-of-things/

[28] Nasajpour M, Pouriyeh S, Parizi RM, Dorodchi M, Valero M, Arabnia HR (2020). Internet of Things for current COVID-19 and future pandemics: An exploratory study. J Healthcare Inf Res.

[29] OTELCO. OTELCO. The Internet of Things in a COVID 19 World: https://www.otelco.com/the-internet-of-things-2021/. 2021.

[30] Parvez N, Chowdhury TH, Urmi SS, Taher KA. Prospects of Internet of Things for Bangladesh. In: 2021 International Conference on Information and Communication Technology for Sustainable Development (ICICT4SD). 2021. p. 481–485.

[31] Pike J, Bogich T, Elwood S, Finnoff DC, Daszak P (2014). Economic optimization of a global strategy to address the pandemic threat. Proc Natl Acad Sci.

[32] Radanliev P, De Roure D (2021). Alternative mental health therapies in prolonged lockdowns: narratives from Covid-19. Heal Technol.

[33] Radanliev P, De Roure D (2021). Epistemological and bibliometric analysis of ethics and shared responsibility—health policy and IoT systems. Sustainability.

[34] Radanliev P, De Roure D, Ani U, Carvalho G (2021). The ethics of shared Covid-19 risks: an epistemological framework for ethical health technology assessment of risk in vaccine supply chain infrastructures. Health Technol.

[35] Rivard S, Huff SL (1988). Factors of success for end-user computing. Commun ACM.

[36] Ryan PJ, Watson RB (2017). Research challenges for the internet of things: what role can or play?. Systems.

[37] Sarker S, Roy K, Afroz F, Pathan ASK. On the Opportunities, Applications, and Challenges of Internet of Things. In: Decentralised Internet of Things (pp. 231–254). Springer, Cham. 2020.

[38] Singh RP, Javaid M, Haleem A, Suman R (2020). Internet of things (IoT) applications to fight against COVID-19 pandemic. Diabetes Metab Syndr.

[39] UNDP. COVID-19 and the SDGs: How the ‘roadmap for humanity’ could be changed by a pandemic. UNDP: https://feature.undp.org/covid-19-and-the-sdgs/. 2020.

[40] Vaske JJ, Beaman J, Sponarski CC (2017). Rethinking internal consistency in Cronbach's alpha. Leis Sci.

[41] Walcott DA. How the Fourth Industrial Revolution can help us beat COVID-19. In World Economic Forum. https://www.weforum. org/agenda/2020/05/how-the-fourth-industrial-revolution-can-help-us-handle-the-threat-of-covid-19. 2020.

[42] WHO. WHO Bangladesh COVID-19 Morbidity and Mortality Weekly Update (MMWU). Coronavirus disease (COVID-2019) Bangladesh situation reports: https://cdn.who.int/media/docs/default-source/searo/bangladesh/covid-19-who-bangladesh-situation-reports/who_covid-19update_57_20210329.pdf?sfvrsn=a3551837_7. 2021.

